# Draft Genome Sequence of Lactobacillus reuteri Strain LR CGMCC 11154, Isolated from the Feces of Healthy Weaned Piglets

**DOI:** 10.1128/MRA.01552-18

**Published:** 2019-01-17

**Authors:** Ting Yu, Xi Yang, Zhilin Wang, Cui Zhu, Jinlong Bei, Li Wang, Wenhua Liu, Qingfeng Zhu, Zongyong Jiang, Zhuang Chen

**Affiliations:** a Key Laboratory of Animal Nutrition and Feed Science (South China) of Ministry of Agriculture, State Key Laboratory of Livestock and Poultry Breeding, Guangdong Public Laboratory of Animal Breeding and Nutrition, Guangdong Key Laboratory of Animal Breeding and Nutrition, Institute of Animal Science, Guangdong Academy of Agricultural Sciences, Guangzhou, China; b Institute of Animal Science, Guangdong Academy of Agricultural Sciences, Guangzhou, China; Indiana University, Bloomington

## Abstract

Lactobacillus reuteri strain LR CGMCC 11154, which was isolated from the feces of healthy weaned piglets, was experimentally proven to be a probiotic bacterium. The whole genome was sequenced on the Illumina Miseq platform to obtain the draft genome, which consists of 120 contigs totaling 1.9 Mbp encoding 1,854 genes.

## ANNOUNCEMENT

**FIG 1:**
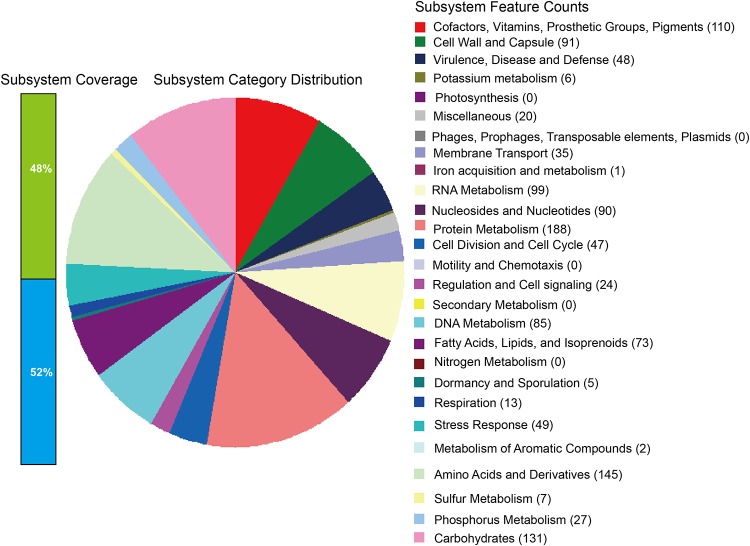
Genes connected to subsystems and their distribution in different categories.

Lactobacillus reuteri is a strain of lactic acid bacterium which belongs to the *Lactobacillus* genus. It is a well-known probiotic bacterium (1, 2). We isolated a strain of the probiotic bacterium from the feces of five healthy 35-day-old weaned piglets (duroc × Landrace × large white) at the Guangdong Academy of Agricultural Sciences. Briefly, about 1 g of feces per piglet was collected by using sterile swabs and stored in sterilized sampling bags with cryopreservation. The swabs were repeatedly and rapidly rinsed with quantitative sterilized phosphate-buffered saline (PBS) and were gradually diluted to 10^6^ cells/ml. Then, a 200-μl liquid sample was coated on a 5-ml Rogosa SL agar plate and cultured anaerobically at 37°C for 48 h. Finally, the strain was identified as Lactobacillus reuteri using 16S rRNA gene sequencing (3) (GenBank accession number KT205306) and named strain LR1 by Z.W. This strain provided excellent health benefits to the weaned piglets (4). At present, this strain is stored in the China General Microbiological Culture Collection Center (CGMCC) under access number 11154. To avoid confusion in the database, we used the unique accession number LR CGMCC 11154 instead of the nonunique name LR1. To further investigate LR CGMCC 11154, we performed its genome sequencing using next-generation sequencing technology. The genomic DNA was extracted using the E.Z.N.A. bacterial DNA kit (product number D3350-01, Omega, USA) following the manufacturer’s instructions, and the library was constructed using the NEBNext Ultra II DNA library prep kit for Illumina (product number E7645L). The whole genome was sequenced on the Illumina MiSeq platform using the 2 × 150-bp MiSeq reagent kit v2 (Illumina, USA). Then, 1,161,442 raw paired-end sequences were generated, and the Q30 quality reached 97%. The raw sequences without trimming because of good quality were used for the *de novo* genome assembly using the IDBA assembler (5) with the default parameters, including precorrection before assembly. The draft genome was assembled into 120 contigs with an *N*_50_ contig size of 30,797 bp and a median coverage depth of 100×. The draft genome of LR CGMCC 11154 is composed of 1,910,900 bp with an average G+C content of 38.6%. Genome annotation was done using the Rapid Annotations using Subsystems Technology (RAST) server (6). A total of 1,854 coding genes and 60 RNAs (including 7 rRNAs and 53 tRNAs) were predicted. Among the coding genes, 1,447 genes (78%) had functional assignments or exhibited homology to proteins with previously known functions, while 407 genes (22%) remained hypothetical proteins. Eighteen subsystem categories in the RAST system were assigned, and the maximum gene count was functionally associated with the amino acids and derivatives (145 coding genes), followed by carbohydrates (131 coding genes) and the category of cofactors, vitamins, prosthetic groups, and pigments (110 coding genes) (Fig. 1). The genome sequence will be useful for genetic modifications of the strain.

### Data availability.

This whole-genome shotgun project of Lactobacillus reuteri strain LR CGMCC 11154 has been deposited at GenBank under the accession number QRDF00000000. The draft genome sequence described in this paper is the first version (QRDF01000000). Raw sequencing reads have been submitted to the Sequence Read Archive (SRA accession number SRR8182721) and are available in the NCBI under BioProject number PRJNA505088 and BioSample number SAMN10410839.

## References

[1] Zhang D, Ji H, Liu H, Wang S, Wang J, Wang Y 2016 Changes in the diversity and composition of gut microbiota of weaned piglets after oral administration of Lactobacillus or an antibiotic. Appl Microbiol Biotechnol 100:1–13. doi:10.1007/s00253-016-7845-5. 27757509

[2] Wang Z, Wang L, Chen Z, Ma X, Yang X, Zhang J, Jiang Z 2016 In vitro evaluation of swine-derived Lactobacillus reuteri: probiotic properties and effects on intestinal porcine epithelial cells challenged with enterotoxigenic Escherichia coli K88. J Microbiol Biotechnol 26:1018–1025. doi:10.4014/jmb.1510.10089. 26907754

[3] Wang ZL, Chen Z, Liao L, Jiang ZY, Wang L, Zheng CT, Wang SF 2012 Optimization of fermentation conditions and selection of the cryoprotectants of Lactobacillus reuteri from piglet. Guangdong Agric Sci 39:151–153. doi:10.16768/j.issn.1004-874x.2012.19.064.

[4] Yi H, Yang G, Xiong Y, Wen X, Wang Z, Yang X, Gao K, Wang L, Jiang Z 2018 Effects of Lactobacillus reuteri LR1 on the growth performance, intestinal morphology and intestinal barrier function in weaned pigs. J Anim Sci 96:2342–2351. doi:10.1093/jas/sky129. 29659876 PMC6095392

[5] Peng Y, Leung HCM, Yiu SM, Chin FYL 2010 IDBA: a practical iterative de Bruijn graph de novo assembler. RECOMB 6044:426–440. doi:10.1007/978-3-642-12683-3_28.

[6] Aziz RK, Bartels D, Best AA, DeJongh M, Disz T, Edwards RA, Formsma K, Gerdes S, Glass EM, Kubal M, Meyer F, Olsen GJ, Olson R, Osterman AL, Overbeek RA, McNeil LK, Paarmann D, Paczian T, Parrello B, Pusch GD, Reich C, Stevens R, Vassieva O, Vonstein V, Wilke A, Zagnitko O 2008 The RAST server: Rapid Annotations using Subsystems Technology. BMC Genomics 9:75. doi:10.1186/1471-2164-9-75. 18261238 PMC2265698

