# Correction for Yu et al., “Draft Genome Sequence of Lactobacillus reuteri Strain LR CGMCC 11154, Isolated from the Feces of Healthy Weaned Piglets”

**DOI:** 10.1128/MRA.00085-19

**Published:** 2019-03-07

**Authors:** Ting Yu, Xi Yang, Zhilin Wang, Cui Zhu, Jinlong Bei, Li Wang, Wenhua Liu, Qingfeng Zhu, Zongyong Jiang, Zhuang Chen

**Affiliations:** a Agro-biological Gene Research Center, Guangdong Academy of Agricultural Sciences, Guangzhou, China; b Key Laboratory of Animal Nutrition and Feed Science (South China) of Ministry of Agriculture, Guangdong Academy of Agricultural Sciences, Guangzhou, China

## AUTHOR CORRECTION

Volume 8, no. 3, e01552-18, 2019, https://doi.org/10.1128/MRA.01552-18. Page 1: The author affiliations should appear as shown in this correction.

